# Development and validation of a nomogram for predicting prolonged intensive care unit stay in patients with acute myocardial infarction and analysis of associated hospitalization costs

**DOI:** 10.3389/fcvm.2026.1900704

**Published:** 2026-08-17

**Authors:** Yufei Hou, Qichao Shi, Shasha Sun, Nan Wang, Yu Wang, Cheng Wang

**Affiliations:** 1 Department of Health Economics, General Hospital of Northern Theater Command, Shenyang, China; 2 Department of Orthopedics, General Hospital of Northern Theater Command, Shenyang, China

**Keywords:** acute myocardial infarction, hospitalization costs, intensive care unit, LASSO regression, nomogram, prolonged length of stay, risk prediction

## Abstract

**Background:**

Prolonged intensive care unit (ICU) stay after acute myocardial infarction (AMI) is associated with increased complications, mortality, and resource use. However, early prediction tools for this population remain lacking. This study aimed to develop and validate a nomogram for predicting prolonged ICU stay in AMI patients and analyze cost differentials.

**Methods:**

This study included 7,571 AMI patients admitted to a tertiary-hospital ICU in China (2019–2025). Prolonged ICU stay was defined as ≥4 days (75th percentile). The cohort was split 8:2 into training and test sets. From 48 candidate variables available within 24 h, Spearman correlation analysis and least absolute shrinkage and selection operator (LASSO) regression selected features. A logistic regression-based nomogram was constructed. Discrimination, calibration, decision curve analysis (DCA), and bootstrap validation (1,000 resamples) were evaluated. Costs were compared between groups and across risk quartiles.

**Results:**

Of 7,571 patients, 2,145 (28.3%) experienced prolonged ICU stay. Nine predictors were retained: pH, lactate dehydrogenase (LDH), blood urea nitrogen (BUN), albumin (ALB), anterior myocardial infarction (Anterior MI), age, partial pressure of carbon dioxide (PaCO_2_), serum chloride (Cl), and mean corpuscular hemoglobin concentration (MCHC). The area under the receiver operating characteristic curve (AUC) was 0.714 (95% confidence interval [CI] 0.700–0.728) and 0.709 (95% CI: 0.679–0.739) in the training and test sets, respectively (DeLong *P* > 0.05). Calibration was satisfactory (Brier score 0.178; Hosmer-Lemeshow *P* > 0.05 in both sets), and DCA confirmed sustained net benefit at 15%–80% threshold probabilities. Bootstrap-corrected C-index was 0.712 (optimism 0.002). At the optimal cutoff, sensitivity, specificity, positive and negative predictive values were 66.8%, 65.3%, 43.2% and 83.3% in the training set, and 64.8%, 68.3%, 44.7% and 83.1% in the test set. Discrimination was robust to the outcome definition (test-set AUC 0.715 under a 90th-percentile threshold).

**Conclusion:**

The nomogram demonstrated moderate discrimination and good calibration. Its operating characteristics do not support use as a standalone tool for individual admission or discharge decisions, but it may aid group-level risk stratification and resource planning, in which predicted risk strata corresponded to graded cost increases. Multicentre external validation is a prerequisite for clinical application.

## 1 Introduction

Acute myocardial infarction (AMI) is the most severe manifestation of coronary atherosclerotic heart disease, characterized by high mortality and substantial long-term disability (1, 2). In China, the incidence and overall disease burden of AMI have continued to escalate owing to population aging, rapid urbanization, and the widespread prevalence of cardiovascular risk factors (3). Notably, the length of stay (LOS) among hospitalized AMI patients in China remains comparatively prolonged by international standards, highlighting considerable room for improvement in critical care resource allocation and management efficiency (4).

Hemodynamic instability, malignant arrhythmias, cardiogenic shock, and concomitant multiorgan dysfunction frequently necessitate admission to the intensive care unit (ICU) for continuous monitoring and organ support in AMI patients (5). Prolonged ICU LOS—commonly defined as exceeding the population median—is not only closely associated with elevated rates of in-hospital complications, increased mortality, and adverse long-term outcomes (6), but also constrains bed turnover, intensifies healthcare resource strain, and substantially inflates hospitalization costs (7). Consequently, early identification of patients at high risk for prolonged ICU stay, coupled with quantification of the incremental economic burden attributable to extended hospitalization, holds significant implications for guiding clinical intervention decisions and optimizing resource allocation (8).

The nomogram is a graphical tool that translates a multivariable regression model into an accessible format for estimating the probability of a given outcome in individual patients (9, 10). When integrated with the least absolute shrinkage and selection operator (LASSO) for variable selection, this approach is particularly well suited to clinical datasets characterized by numerous candidate predictors and potential multicollinearity, enabling the construction of parsimonious and clinically deployable prediction models while effectively mitigating overfitting (11). However, the majority of existing prediction studies on ICU LOS have relied on public databases such as the Medical Information Mart for Intensive Care (MIMIC), which predominantly comprise Western populations, and lack model development and validation based on real-world data from Chinese cohorts (12). Furthermore, prior studies have largely confined their scope to predicting LOS *per se*, with few integrating the risk prediction of prolonged ICU stay with hospitalization cost structure analysis, thereby precluding a comprehensive evidence base that jointly addresses clinical effectiveness and economic efficiency.

To address these gaps, the present study utilized ICU admission data of AMI patients from a tertiary teaching hospital in northeastern China to accomplish two objectives: (1) to develop and validate a LASSO–logistic regression-based nomogram for predicting prolonged ICU LOS; and (2) to compare the hospitalization cost structure between patients with and without prolonged ICU stay, thereby quantifying the incremental economic burden associated with extended hospitalization. By establishing an integrated “risk prediction–economic evaluation” analytical framework, this study aims to provide a quantitative tool and evidence-based reference for early risk stratification, rational ICU resource allocation, and hospitalization cost management in AMI patients.

## 2 Methods

### 2.1 Data source

This study was a retrospective cohort study based on a single-center electronic medical record (EMR) system. Data were extracted from hospitalization records of patients admitted to a large tertiary teaching hospital in China between 2019 and 2025. The database encompassed demographic information, admission and discharge diagnoses, mode of admission, vital signs, first laboratory test results obtained after admission (including complete blood count [CBC], serum biochemistry, and arterial blood gas [ABG] analysis), comorbidity profiles, and itemized hospitalization cost records. This study was reviewed and approved by the Ethics Committee of the General Hospital of Northern Theater Command (approval no. Y2026-181) and was conducted in accordance with the principles of the Declaration of Helsinki. Given the retrospective design and the use of de-identified data, the requirement for informed consent was waived by the ethics committee.

### 2.2 Inclusion and exclusion criteria

The inclusion criteria were as follows: (1) age ≥18 years; (2) a primary discharge diagnosis of AMI (International Classification of Diseases, 10th Revision [ICD-10] codes: I21.0–I21.9); and (3) ICU admission during the index hospitalization with complete records of both ICU admission and discharge timestamps.

The exclusion criteria were: (1) missing ICU LOS data; and (2) implausible ICU LOS values, defined as ≤0 days or >90 days, to exclude data entry errors and extreme long-stay outliers. After applying these criteria, a final analytic cohort of 7,571 patients was established, of whom 2,145 (28.33%) experienced prolonged ICU stay (the positive event). Based on the criterion of at least 10 events per variable (EPV ≥10), the final model containing 9 predictors required approximately 90 events, whereas the initial screening of 48 candidate variables required approximately 480 events; the observed number of events (*n* = 2,145) exceeded both thresholds, confirming adequate sample size (13).

### 2.3 Variable definitions

#### 2.3.1 Outcome variable

The primary outcome was prolonged ICU stay. Given the right-skewed distribution of ICU LOS (measured in whole days), the 75th percentile of the analytic cohort was selected as the threshold, corresponding to 4 days. Patients with an ICU LOS ≥4 days were classified as having prolonged stay (coded as 1), and the remainder as non-prolonged (coded as 0), yielding a prolonged-stay proportion of 28.33%. The “≥” operator was adopted to capture patients whose LOS fell exactly at the threshold (using “>” would have reduced the prolonged-stay proportion to 14.69%).

### 2.3.2 Predictor variables

The prediction model was designed to estimate the probability of subsequent prolonged ICU stay based on information available from admission through the first 24 h after ICU entry. The prediction time point was set at 24 h post-ICU admission (i.e., the end of the first ICU day), by which time initial laboratory results would have been obtained—consistent with the data window used by widely adopted ICU prognostic scoring systems. A total of 48 candidate predictors were organized by clinical domain as follows: (a) demographics and anthropometrics: Age, Sex, and body mass index (BMI); (b) admission information: AdmissionType (mode of admission); (c) infarction characteristics: Anterior MI (anterior wall involvement), infarction subtype (ST-segment elevation myocardial infarction [STEMI] vs. non-ST-segment elevation myocardial infarction [NSTEMI]), and Surgery (whether a surgical or interventional procedure was performed prior to ICU admission)—Anterior MI and infarction subtype were determined from the acute diagnosis text at admission; (d) comorbidity burden: Charlson Comorbidity Index (CCI); (e) vital signs: systolic blood pressure (SBP) and diastolic blood pressure (DBP) at admission; and (f) laboratory parameters (all first values obtained within 24 h of ICU admission), comprising CBC indices (white blood cell [WBC] count, neutrophil percentage [NEUT%], absolute neutrophil count [ANC], absolute lymphocyte count [ALC], monocyte percentage [MONO%], absolute monocyte count [AMC], basophil count [BASO], red blood cell [RBC] count, hemoglobin [Hb], platelet count [PLT], and mean corpuscular hemoglobin concentration [MCHC]), serum biochemistry (albumin [ALB], total protein [TP], globulin [GLOB], alanine aminotransferase [ALT], aspartate aminotransferase [AST], total bilirubin [TBIL], alkaline phosphatase [ALP], serum creatinine [SCr], blood urea nitrogen [BUN], serum potassium [K], serum sodium [Na], serum chloride [Cl], total carbon dioxide [TCO_2_], creatine kinase [CK], creatine kinase-MB isoenzyme [CK-MB], lactate dehydrogenase [LDH], fasting plasma glucose [FPG], glycated hemoglobin [HbA1c], triglycerides [TG], low-density lipoprotein cholesterol [LDL-C], high-density lipoprotein cholesterol [HDL-C], and total cholesterol [TC]), and ABG parameters (pH, partial pressure of carbon dioxide [PaCO_2_], partial pressure of oxygen [PaO_2_], arterial oxygen saturation [SaO_2_], and Lactate).

#### 2.3.3 Cost variables

For the supplementary cost analysis, the following variables were extracted: total hospitalization costs, ICU-specific costs, and itemized cost categories including pharmaceutical costs, diagnostic examination fees, laboratory test fees, surgical/procedural fees, medical consumable costs, therapeutic procedure fees, bed charges, nursing care fees, and miscellaneous costs (all denominated in Chinese yuan [CNY]).

### 2.4 Data processing

All continuous variables first underwent forced numeric conversion, whereby non-numeric entries (e.g., character strings) embedded within numeric fields were recoded as missing. Subsequently, clinically implausible values were cleaned by applying pre-specified, clinically reasonable upper and lower bounds for each continuous variable; values falling outside these bounds were set to missing. To mitigate the influence of residual extreme values on model estimation, all continuous variables were winsorized at the 0.5th and 99.5th percentiles—that is, values below the 0.5th or above the 99.5th percentile were truncated to the corresponding percentile value. The proportion of missing data was then calculated for each candidate variable, and variables with a missing rate exceeding 30% were excluded; patient identifiers, outcome variables, and cost variables were exempt from this exclusion. The missing data proportions for all candidate variables are presented in Supplementary Table S1.

Prior to missing data imputation, the analytic cohort was randomly split into a training set and a test set at an 8:2 ratio, stratified by the primary outcome variable (random seed fixed at 42), to ensure comparable event rates across both sets and to prevent data leakage during subsequent imputation and modeling. Missing values were handled using multiple imputation by chained equations (MICE), implemented via the IterativeImputer in scikit-learn with Bayesian ridge regression as the base estimator (maximum of 10 iterations; median used for initial imputation). The imputation model was fitted exclusively on the training set, and the fitted model was then applied to transform the test set, thereby strictly preventing information leakage from the test set into the training process. Categorical variables were encoded before imputation and decoded afterward; imputed values for binary variables were rounded and constrained to the 0/1 range.

### 2.5 Feature selection

Feature selection was performed exclusively on the training set to avoid test-set information leakage. Pairwise Spearman rank correlation coefficients were first computed among all 48 candidate variables. This screen was applied only to pairs of mutually collinear predictors: where |*ρ*| ≥ 0.8 between two variables, one was retained and the other removed, with the correlation with the outcome used solely as the rule for deciding which member of the pair to retain. Variables not strongly collinear with any other predictor were carried forward irrespective of their marginal association with the outcome. Twelve collinear pairs were identified on this basis and ten variables were removed, leaving 38 variables to enter the LASSO. The threshold of |*ρ*| ≥ 0.8 follows convention and is not derived from a formal criterion (Supplementary Table S2).

The remaining variable set was then subjected to LASSO-penalized binary logistic regression for variable selection. The optimal penalty parameter *λ* was determined via 10-fold cross-validation, and the more conservative *λ*_1_ₛₑ (i.e., the largest *λ* within one standard error of the minimum cross-validated deviance) was ultimately adopted to yield a more parsimonious and robust prediction model. Variables whose coefficients were shrunk to zero were excluded, and those with non-zero coefficients were retained for subsequent model development (14, 15).

### 2.6 Model development and nomogram construction

Prediction model development and nomogram construction were performed in R software (version [4.5.1]) using the rms, pROC, ResourceSelection, Hmisc, and ggplot2 packages, and reporting adhered to the Transparent Reporting of a multivariable prediction model for Individual Prognosis Or Diagnosis (TRIPOD) guidelines. The variables retained after LASSO selection (at *λ*_1_ₛₑ) were entered into a multivariable logistic regression model fitted on the training set. Regression coefficients, odds ratios (ORs) with 95% confidence intervals (CIs; Wald-based), and *P* values were estimated on the training set. ORs were reported per clinically meaningful unit increment for each variable (e.g., per 0.1-unit increase in pH; per 100 U/L increase in LDH). The test set was used solely for subsequent internal validation and did not contribute to coefficient estimation or variable selection. Based on the fitted multivariable logistic regression model, a nomogram was constructed for individualized prediction of the probability of prolonged ICU stay.

### 2.7 Statistical analysis

Descriptive statistics and between-group comparisons. Baseline characteristics were described for the full analytic cohort (*N* = 7,571) and compared between the prolonged and non-prolonged ICU stay groups, with the number of missing observations reported for each variable. Continuous variables were assessed for normality using the Shapiro–Wilk test; approximately normally distributed variables were expressed as mean ± standard deviation (SD) and compared using the independent-samples *t*-test (with Welch's correction when variances were unequal), whereas non-normally distributed variables were expressed as median (interquartile range [IQR]) and compared using the Mann–Whitney *U*-test. Categorical variables were expressed as frequency (percentage) and compared using the chi-squared (*χ*^2^) test, with Fisher's exact test applied when any expected cell count in a 2 × 2 contingency table was less than 5.

As a supplementary analysis of clinical outcomes, an exploratory cost analysis was conducted. Given the skewed distribution of cost variables, all costs were described as median (IQR). Costs were first compared between the prolonged and non-prolonged ICU stay groups using the Mann–Whitney *U*-test, and the median difference and fold change between groups were calculated. The full cohort was then stratified into four risk groups (Q1–Q4) based on the quartiles of the nomogram-predicted probability, and total hospitalization costs and ICU-specific costs were compared across risk strata using the Kruskal–Wallis test.

Model performance evaluation and internal validation. Discrimination was assessed using the area under the receiver operating characteristic (ROC) curve (AUC) with its 95% CI, and the DeLong test was used to compare AUCs between the training and test sets. Calibration was evaluated using calibration plots, calibration intercept and slope, Brier score, and the Hosmer–Lemeshow goodness-of-fit test (with data divided into 10 groups). Clinical utility was assessed via decision curve analysis (DCA), which compared the net benefit of the model against the “treat-all” and “treat-none” reference strategies across a range of threshold probabilities. In addition, the optimal probability cutoff was determined based on the Youden index, and the corresponding sensitivity, specificity, positive predictive value (PPV), negative predictive value (NPV), and accuracy were calculated. All of the above metrics were reported separately for the training and test sets. Internal validation was further performed using 1,000 bootstrap resamples to estimate the degree of model optimism, and an optimism-corrected C-index and calibration curve were generated. Variance inflation factors were calculated for all predictors in the final model, with VIF ≥5 taken to indicate problematic collinearity. To assess whether a more parsimonious model would perform comparably, nested models were fitted by adding predictors in descending order of their Wald *χ*^2^ statistic and compared with the full model using the DeLong test for test-set AUC, the likelihood-ratio test, and the Akaike information criterion.

### 2.8 Sensitivity analysis

To assess whether the model depended on the specific definition of the outcome, we repeated the entire analytic pipeline—Spearman correlation screening, LASSO selection at *λ*1se, multivariable logistic regression, and bootstrap internal validation—using a more stringent definition of prolonged ICU stay based on the 90th percentile of ICU LOS (≥5 days) rather than the 75th percentile (≥4 days). The same 8:2 training/test split, random seed, and preprocessing steps were used, so that the outcome definition was the only element that differed.

Statistical software and significance level. Data preprocessing, missing data imputation, and baseline descriptive statistics were performed using Python 3.9.13 (pandas, NumPy, SciPy, and scikit-learn). Feature selection, model development, model validation, and cost analysis were conducted in R 4.5.1. All hypothesis tests were two-sided, and *P* < 0.05 was considered statistically significant.

## 3 Results

### 3.1 Study population and baseline characteristics

A total of 7,571 AMI patients were included in this study. The median ICU LOS for the full cohort was 3.0 (IQR 2.0–4.0) days. Using the 75th percentile (4 days) as the cutoff, 2,145 patients (28.3%) were classified as having prolonged ICU stay (≥4 days) and 5,426 (71.7%) as non-prolonged. Baseline characteristics of the two groups are compared in Table 1.

**Table 1:** Baseline characteristics of AMI patients stratified by ICU length of stay.

Characteristic	Overall (*N* = 7,571)	Prolonged ICU LOS (*n* = 2,145)	Non-prolonged (*n* = 5,426)	*P* value
Demographics				
Age (years), median (IQR)	62 (53–69)	63 (55–71)	61 (52–69)	<0.001
Sex, *n* (%)				0.021
Male	5,812 (76.8)	1,608 (75.0)	4,204 (77.5)	
Female	1,759 (23.2)	537 (25.0)	1,222 (22.5)	
BMI (kg/m^2^), median (IQR)	25.2 (23.0–27.7)	24.9 (22.7–27.3)	25.3 (23.1–27.7)	<0.001
Admission				
Admission type, *n* (%)				0.07
Emergency	7,133 (94.2)	2,038 (95.0)	5,095 (93.9)	
Outpatient	438 (5.8)	107 (5.0)	331 (6.1)	
Myocardial infarction				
Anterior MI, *n* (%)				<0.001
Yes	2,966 (39.2)	1,024 (47.7)	1,942 (35.8)	
No	4,605 (60.8)	1,121 (52.3)	3,484 (64.2)	
STEMI, *n* (%)				0.011
STEMI	5,456 (72.8)	1,580 (74.9)	3,876 (71.9)	
Non-STEMI	2,042 (27.2)	530 (25.1)	1,512 (28.1)	
Surgery, *n* (%)				0.411
Yes	6,792 (89.7)	1,914 (89.2)	4,878 (89.9)	
No	779 (10.3)	231 (10.8)	548 (10.1)	
Comorbidity				
CCI, median (IQR)	3 (2–4)	4 (3–4)	3 (2–4)	<0.001
Vital signs				
SBP (mmHg), median (IQR)	134 (121–146)	131 (119–144)	135 (122–148)	<0.001
DBP (mmHg), median (IQR)	83 (76–91)	82 (75–90)	84 (76–92)	<0.001
Complete blood count				
WBC (×10^9^/L), median (IQR)	10.40 (8.30–13.00)	10.90 (8.70–13.60)	10.30 (8.20–12.70)	<0.001
NEUT%, median (IQR)	83.90 (75.70–89.50)	84.60 (76.60–89.80)	83.60 (75.30–89.30)	0.002
ANC (×10^9^/L), median (IQR)	8.68 (6.56–11.08)	9.10 (6.72–11.80)	8.50 (6.50–10.83)	<0.001
ALC (×10^9^/L), median (IQR)	1.14 (0.80–1.66)	1.11 (0.76–1.59)	1.16 (0.80–1.70)	<0.001
MONO%, median (IQR)	5.00 (2.00–6.90)	5.20 (2.50–7.20)	4.90 (1.80–6.72)	<0.001
AMC (×10^9^/L), median (IQR)	0.48 (0.20–0.72)	0.53 (0.28–0.80)	0.46 (0.19–0.70)	<0.001
BASO (×10^9^/L), median (IQR)	0.10 (0.10–0.30)	0.10 (0.10–0.30)	0.10 (0.10–0.30)	<0.001
RBC (×10^12^/L), median (IQR)	4.48 (4.08–4.88)	4.43 (4.00–4.86)	4.50 (4.12–4.89)	<0.001
Hb (g/L), median (IQR)	139.00 (126.00–151.00)	137.00 (123.00–150.00)	139.00 (127.00–151.00)	<0.001
PLT (×10^9^/L), median (IQR)	218.00 (181.00–257.00)	217.00 (179.00–257.00)	218.00 (182.00–257.00)	0.356
MCHC (g/L), median (IQR)	333.00 (327.00–338.00)	332.00 (326.00–337.00)	333.00 (328.00–338.00)	<0.001
Biochemistry				
ALB (g/L), median (IQR)	38.00 (35.30–40.60)	37.30 (34.50–40.00)	38.20 (35.70–40.80)	<0.001
TP (g/L), median (IQR)	64.60 (60.60–68.60)	63.70 (59.80–68.40)	64.90 (61.00–68.80)	<0.001
GLOB (g/L), median (IQR)	26.50 (24.00–29.20)	26.70 (24.00–29.40)	26.50 (24.00–29.10)	0.120
ALT (U/L), median (IQR)	39.20 (24.80–61.00)	48.23 (29.00–78.91)	36.43 (23.88–55.44)	<0.001
AST (U/L), median (IQR)	125.05 (50.73–250.26)	182.70 (68.69–351.08)	109.35 (46.33–212.46)	<0.001
TBIL (μmol/L), median (IQR)	10.90 (8.10–15.00)	11.30 (8.60–15.80)	10.80 (8.00–14.60)	<0.001
ALP (U/L), median (IQR)	75.79 (63.05–91.47)	75.91 (63.10–92.32)	75.76 (63.01–90.98)	0.407
SCr (μmol/L), median (IQR)	73.00 (61.47–87.08)	77.44 (64.61–95.17)	71.13 (60.30–84.14)	<0.001
BUN (mmol/L), median (IQR)	5.79 (4.65–7.36)	6.60 (5.12–8.61)	5.58 (4.51–6.89)	<0.001
K (mmol/L), median (IQR)	4.11 (3.86–4.37)	4.12 (3.86–4.40)	4.10 (3.87–4.35)	0.125
Na (mmol/L), median (IQR)	139.00 (137.30–140.70)	138.90 (137.00–140.70)	139.10 (137.40–140.70)	0.004
Cl (mmol/L), median (IQR)	104.30 (102.10–106.40)	103.80 (101.40–106.20)	104.50 (102.40–106.40)	<0.001
TCO_2_ (mmol/L), median (IQR)	23.70 (21.70–26.00)	23.60 (21.60–26.10)	23.70 (21.80–25.90)	0.713
CK (U/L), median (IQR)	1,044.00 (357.00–2,072.50)	1,372.00 (409.00–2,620.00)	960.50 (346.00–1,854.50)	<0.001
CK-MB (U/L), median (IQR)	100.60 (35.52–208.97)	139.45 (39.18–292.25)	90.65 (34.00–178.07)	<0.001
LDH (U/L), median (IQR)	534.00 (317.00–878.25)	714.50 (418.00–1,167.75)	479.00 (292.00–769.00)	<0.001
FPG (mmol/L), median (IQR)	7.17 (5.86–9.54)	7.51 (6.00–10.36)	7.04 (5.82–9.28)	<0.001
HbA1c (%), median (IQR)	6.07 (5.66–7.33)	6.10 (5.69–7.48)	6.05 (5.65–7.28)	0.012
TG (mmol/L), median (IQR)	1.33 (0.95–1.92)	1.30 (0.94–1.82)	1.34 (0.95–1.96)	0.021
LDL-C (mmol/L), median (IQR)	2.88 (2.31–3.52)	2.80 (2.25–3.43)	2.92 (2.34–3.55)	<0.001
HDL-C (mmol/L), median (IQR)	1.14 (0.97–1.34)	1.15 (0.97–1.35)	1.14 (0.97–1.33)	0.242
TC (mmol/L), median (IQR)	4.79 (4.06–5.63)	4.68 (3.96–5.51)	4.84 (4.10–5.66)	<0.001
Arterial blood gas				
pH, median (IQR)	7.39 (7.37–7.42)	7.40 (7.37–7.43)	7.39 (7.37–7.41)	<0.001
PaCO_2_ (mmHg), median (IQR)	38.40 (35.30–41.30)	37.00 (33.70–40.00)	38.90 (36.10–41.70)	<0.001
PaO_2_ (mmHg), median (IQR)	97.20 (82.00–119.00)	94.30 (78.80–116.00)	98.80 (83.50–120.00)	<0.001
SaO_2_ (%), median (IQR)	97.10 (95.60–98.30)	96.90 (95.10–98.20)	97.20 (95.80–98.30)	<0.001
Lactate (mmol/L), median (IQR)	2.20 (1.70–2.90)	2.20 (1.70–3.00)	2.20 (1.70–2.80)	0.049
ICU length of stay				
ICU LOS (days), median (IQR)	3 (2–4)	5 (4–6)	2.0 (2–3)	<0.001

Compared with the non-prolonged group, patients in the prolonged group were older (median 63 [IQR 55–71] vs. 61 [IQR 52–69] years; *P* < 0.001), had a slightly lower proportion of males (75.0% vs. 77.5%; *P* = 0.021), and had a marginally lower BMI (*P* < 0.001). The prolonged group also exhibited a higher prevalence of Anterior MI (47.7% vs. 35.8%; *P* < 0.001) and STEMI (74.9% vs. 71.9%; *P* = 0.011), a higher CCI (median 4 [IQR 3–4] vs. 3 [IQR 2–4]; *P* < 0.001), and lower SBP and DBP at admission (both *P* < 0.001). AdmissionType and Surgery did not differ significantly between the two groups (both *P* > 0.05).

Regarding laboratory parameters, the prolonged group demonstrated more pronounced inflammatory activation and myocardial injury: WBC, ANC, and AMC were higher, while ALC was lower (all *P* < 0.001). Myocardial necrosis markers, including CK (1,372 vs. 960 U/L), CK-MB (139 vs. 91 U/L), and LDH (714 vs. 479 U/L), were all significantly elevated in the prolonged group (all *P* < 0.001). Furthermore, the prolonged group exhibited worse renal function (SCr 77 vs. 71 μmol/L; BUN 6.6 vs. 5.6 mmol/L), lower ALB (37.3 vs. 38.2 g/L), and higher FPG (7.5 vs. 7.0 mmol/L) (all *P* < 0.001). ABG analysis revealed lower PaO_2_, SaO_2_, and PaCO_2_ in the prolonged group (all *P* < 0.001).

### 3.2 Feature selection

**Figure 1:**
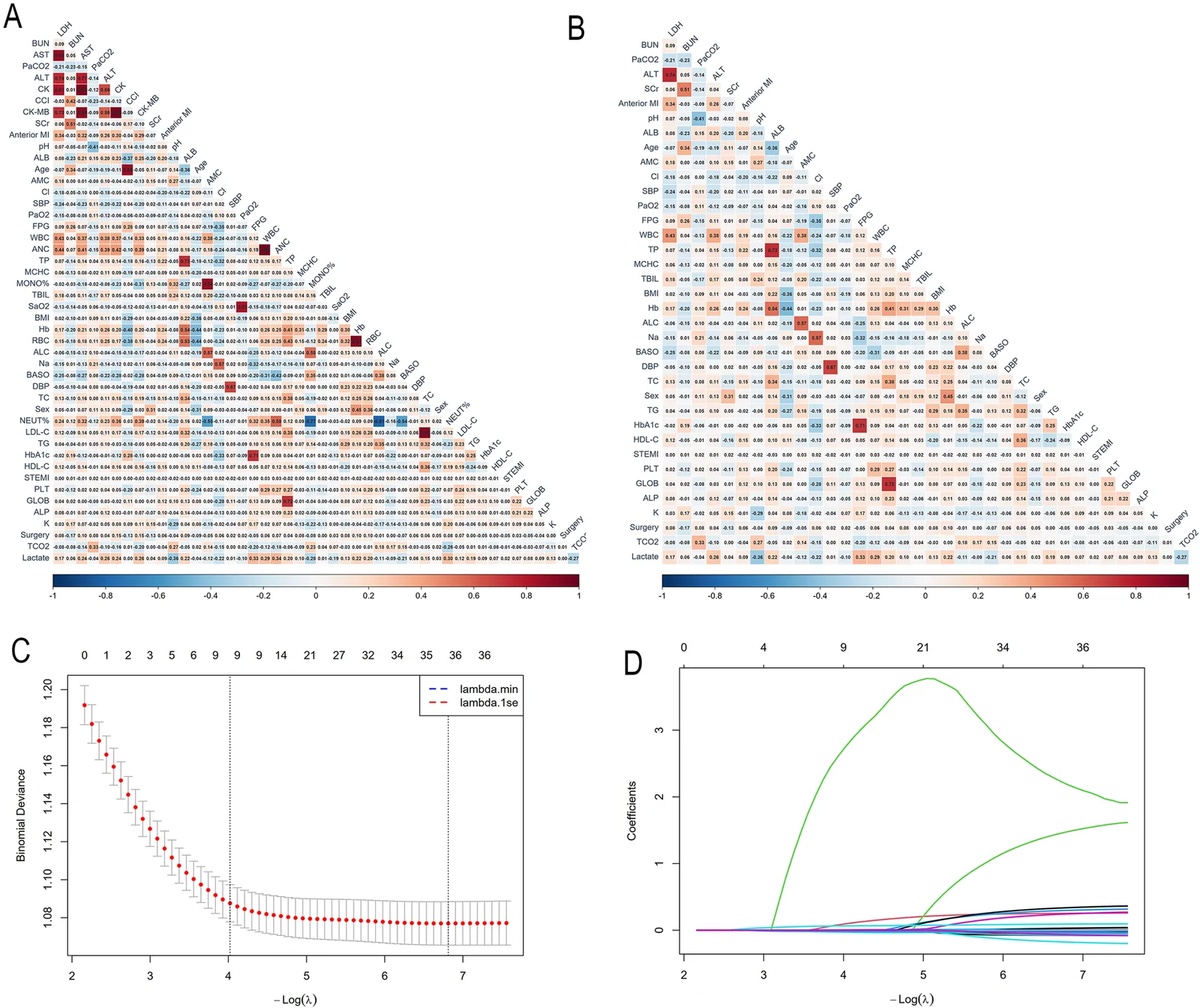
Feature selection for predicting prolonged ICU stay. (A) Spearman correlation matrix of all 48 candidate variables. (B) Spearman correlation matrix of the 38 variables retained after removing highly correlated pairs (|*ρ*| ≥ 0.8). (C) Selection of the optimal penalty parameter (*λ*) in the LASSO regression via 10-fold cross-validation; the dotted vertical lines indicate *λ*_min and *λ*_1se, with the more conservative *λ*_1se adopted for the final model. (D) LASSO coefficient profiles of the candidate variables as a function of Log(*λ*).

A total of 48 candidate variables were initially considered, including Age, Sex, BMI, SBP, DBP, Anterior MI, STEMI, Surgery, AdmissionType, CCI, WBC, NEUT%, ANC, ALC, MONO%, AMC, BASO, RBC, Hb, PLT, MCHC, ALB, TP, GLOB, ALT, AST, TBIL, ALP, SCr, BUN, K, Na, Cl, TCO_2_, CK, CK-MB, LDH, FPG, HbA1c, TG, LDL-C, HDL-C, TC, pH, PaCO_2_, PaO_2_, SaO_2_, and Lactate. Spearman correlation analysis identified 12 pairs of highly correlated variables (|*ρ*| ≥ 0.8). Following the principle of retaining the variable with the stronger correlation with the primary outcome, 10 variables were removed—CCI, ANC, NEUT%, MONO%, RBC, CK, CK-MB, AST, LDL-C, and SaO_2_—leaving 38 variables for LASSO selection (Figures 1A,B). After 10-fold cross-validated LASSO regression (at *λ*_1_ₛₑ), 9 variables retained non-zero coefficients and were included in the final prediction model: pH, Anterior MI, BUN, ALB, PaCO_2_, Cl, MCHC, Age, and LDH (Figures 1C,D).

### 3.3 Model development and nomogram

The 9 variables selected by LASSO were entered into a multivariable logistic regression model, and all achieved statistical significance (Table 2). Among the risk-increasing predictors, pH (per 0.1-unit increase; OR = 1.54, 95% CI: 1.31–1.81), LDH (per 100 U/L increase; OR = 1.12, 95% CI: 1.10–1.13), BUN (per 1 mmol/L increase; OR = 1.08, 95% CI: 1.06–1.10), Anterior MI (OR = 1.31, 95% CI: 1.15–1.50), and Age (per 1-year increase; OR = 1.01, 95% CI: 1.00–1.02) were independently associated with an elevated risk of prolonged ICU stay. Conversely, ALB (per 1 g/L increase; OR = 0.95, 95% CI: 0.93–0.97), Cl (per 1 mmol/L increase; OR = 0.97, 95% CI: 0.95–0.99), PaCO_2_ (per 1 mmHg increase; OR = 0.98, 95% CI: 0.96–0.99), and MCHC (per 1 g/L increase; OR = 0.98, 95% CI: 0.98–0.99) were independently associated with a reduced risk.

**Table 2:** Multivariable logistic regression analysis of predictors for prolonged ICU stay in AMI patients.

Variable	*β* (SE)	Wald *χ*^2^	OR (95% CI)	*P* value
Intercept	−23.037 (6.640)	12.04	—	<0.001
pH (per 0.1 unit)	0.432 (0.082)	27.95	1.54 (1.31–1.81)	<0.001
BUN (per 1 mmol/L)	0.077 (0.011)	46.18	1.08 (1.06–1.10)	<0.001
ALB (per 1 g/L)	−0.053 (0.009)	35.25	0.95 (0.93–0.97)	<0.001
PaCO_2_ (per 1 mmHg)	−0.025 (0.008)	10.82	0.98 (0.96–0.99)	0.001
Cl (per 1 mmol/L)	−0.032 (0.009)	11.42	0.97 (0.95–0.99)	<0.001
MCHC (per 1 g/L)	−0.017 (0.004)	20.42	0.98 (0.98–0.99)	<0.001
Age (per 1 year)	0.010 (0.003)	12.59	1.01 (1.00–1.02)	<0.001
LDH (per 100 U/L)	0.112 (0.007)	225.97	1.12 (1.10–1.13)	<0.001
Anterior MI (yes vs. no)	0.272 (0.068)	16.09	1.31 (1.15–1.50)	<0.001

**Figure 2:**
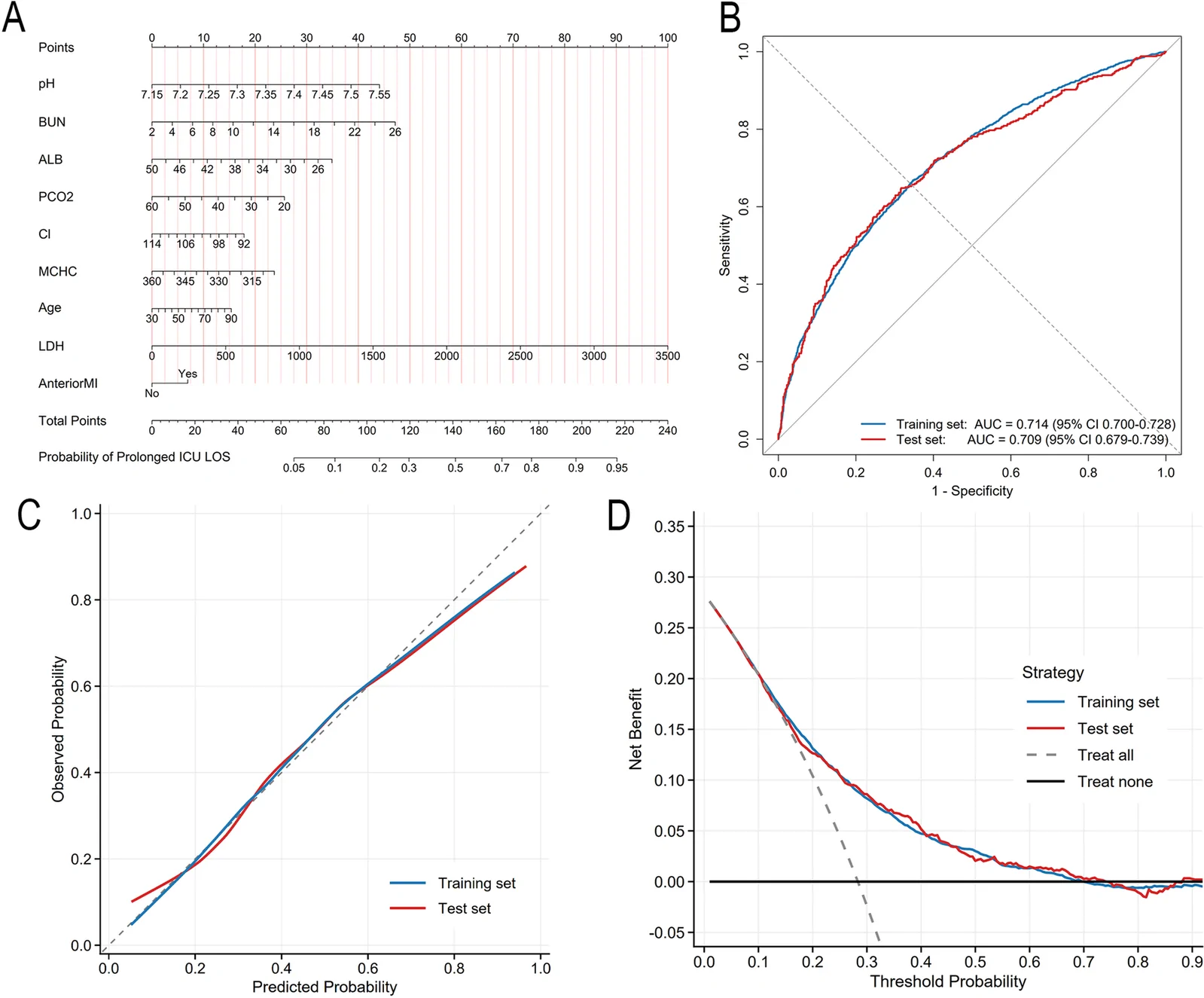
Construction and performance evaluation of the nomogram. **(A)** Nomogram for predicting the probability of prolonged ICU stay based on nine predictors (pH, BUN, ALB, PaCO_2_, Cl, MCHC, Age, LDH, and Anterior MI). **(B)** Receiver operating characteristic (ROC) curves for the training and test sets. **(C)** Decision curve analysis (DCA) showing the net benefit across threshold probabilities for the training and test sets, compared with the “treat-all” and “treat-none” strategies. **(D)** Calibration curves for the training and test sets.

The nomogram is used as follows. For each predictor, a vertical line is drawn upward from the patient's value to the “Points” axis at the top of the figure to obtain the corresponding score. The scores for all nine predictors are then summed to give the total points, which is located on the “Total Points” axis; a vertical line drawn downward from this position yields the predicted probability of prolonged ICU stay. For example, a 75-year-old patient with an anterior MI, pH 7.50, BUN 15 mmol/L, albumin 28 g/L, PaCO_2_ 30 mmHg, chloride 96 mmol/L, MCHC 315 g/L and LDH 800 U/L accumulates 182 total points, corresponding to a predicted probability of 86%. As this substantially exceeds the Youden-derived cutoff of 0.264, the patient would be classified as high risk for prolonged ICU stay. Based on the fitted multivariable logistic regression model, a nomogram was constructed for individualized prediction of the probability of prolonged ICU stay (Figure 2A).

### 3.4 Model performance evaluation and internal validation

Regarding discrimination, the AUC was 0.714 (95% CI: 0.700–0.728) in the training set and 0.709 (95% CI: 0.679–0.739) in the test set, with no statistically significant difference between the two (DeLong test), indicating satisfactory generalizability (Figure 2B). Regarding calibration, the Brier score was 0.178 in both the training and test sets, and the Hosmer–Lemeshow test was non-significant in both sets (training *P* = 0.918; test *P* = 0.356), demonstrating good agreement between predicted probabilities and observed event rates (Figure 2C). DCA showed that the model yielded a consistently positive net benefit over the “treat-all” and “treat-none” reference strategies across threshold probabilities of approximately 15%–80% (Figure 2D).

Based on the Youden index, the optimal cutoff values were 0.264 and 0.279 for the training and test sets, respectively. At these cutoffs, sensitivity was 66.8% and 64.8%, specificity 65.3% and 68.3%, PPV 43.2% and 44.7%, and NPV 83.3% and 83.1%, in the training and test sets respectively. Internal validation with 1,000 bootstrap resamples yielded an optimism-corrected C-index of 0.712 (95% CI: 0.699–0.727) with an optimism estimate of only 0.002, and an optimism-corrected calibration slope of 0.990 (95% CI: 0.890–1.093), indicating minimal overfitting and robust model performance (Supplementary Table S3).

Variance inflation factors for all nine predictors were low (range 1.09–1.34), indicating no problematic collinearity (Supplementary Table S4). In nested models fitted by adding predictors in descending order of Wald *χ*^2^, a four-variable model comprising LDH, BUN, albumin and pH achieved a test-set AUC of 0.710 (95% CI: 0.680–0.740), statistically indistinguishable from the full nine-variable model (0.709; DeLong *P* = 0.891); the remaining predictors nonetheless improved model fit (likelihood-ratio test *P* < 0.001; AIC 6,573.9 to 6,511.5) (Supplementary Table S5).

### 3.5 Cost analysis

**Figure 3:**
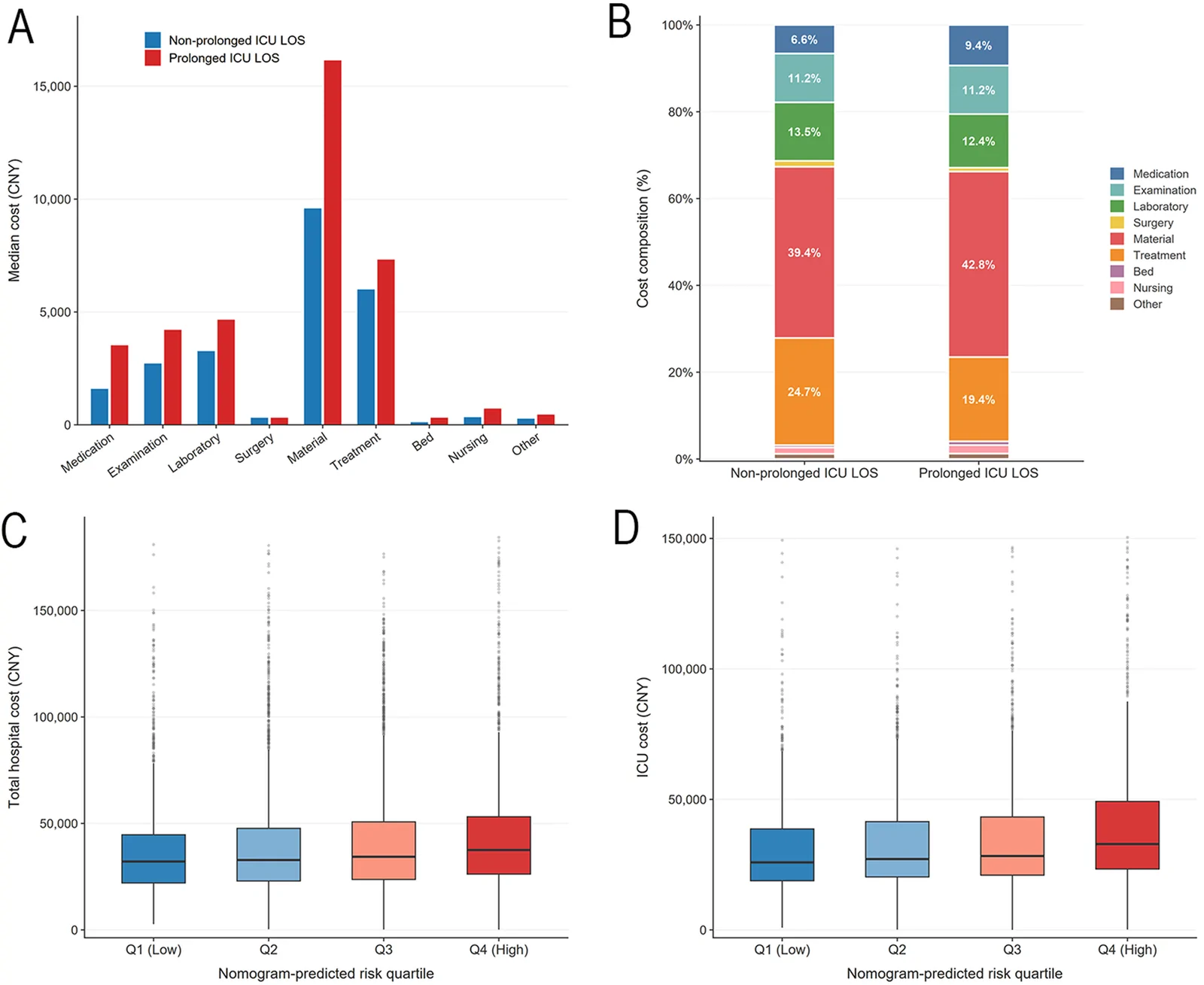
Hospitalization cost analysis. **(A)** Comparison of median costs across itemized cost categories between the prolonged and non-prolonged ICU stay groups. **(B)** Cost composition (%) of each itemized category in the two groups. **(C)** Total hospitalization costs stratified by nomogram-predicted risk quartiles (Q1–Q4). **(D)** ICU-specific costs stratified by nomogram-predicted risk quartiles (Q1–Q4).

Total hospitalization costs and ICU-specific costs were both significantly higher in the prolonged group than in the non-prolonged group (both *P* < 0.001). The median total hospitalization cost in the prolonged group was CNY 45,457 (IQR CNY 30,543–64,131), which was CNY 15,049 higher than the non-prolonged group (CNY 30,408 [IQR CNY 22,160–44,392]), representing a 1.49-fold difference. The median ICU-specific cost was CNY 40,494 (IQR CNY 26,794–56,436) in the prolonged group vs. CNY 25,519 (IQR CNY 19,352–38,147) in the non-prolonged group, a difference of CNY 14,975 (1.59-fold). All itemized cost categories were significantly elevated in the prolonged group, with bed charges (2.50-fold), pharmaceutical costs (2.20-fold), and nursing care fees (2.08-fold) showing the greatest proportional increases. Medical consumable costs constituted the largest expenditure category in both groups (39.4% in the non-prolonged group and 42.8% in the prolonged group) and exhibited the greatest absolute difference of CNY 6,557 (1.68-fold) (Supplementary Table S6; Figures 3A,B).

When stratified by nomogram-predicted probability quartiles, both total hospitalization costs and ICU-specific costs demonstrated a monotonically increasing trend with ascending risk strata (Supplementary Table S7; Figures 3C,D): the median total hospitalization cost rose from CNY 32,278 in Q1 (lowest risk) to CNY 38,087 in Q4 (highest risk), and median ICU-specific costs increased from CNY 25,863 to CNY 33,590 (Kruskal–Wallis test *P* < 0.001). Moreover, the observed prolonged ICU stay rates across risk strata (Q1–Q4: 12.9%, 18.3%, 30.9%, and 51.2%) closely matched the nomogram-predicted probabilities (12.9%, 20.4%, 29.3%, and 50.8%), further corroborating the calibration performance of the model across the full cohort.

To examine whether the model captured severity beyond length of stay itself, costs were additionally compared across predicted-risk quartiles within the non-prolonged group (*n* = 5,426), in whom ICU LOS was by definition short. Median total cost did not increase monotonically across quartiles (CNY 29,574, 31,133, 31,081 and 30,070 for Q1–Q4; Q4/Q1 ratio 1.02), and median ICU cost rose only marginally (CNY 24,082 to 26,521; ratio 1.10). Although Kruskal–Wallis tests were significant for both (*P* < 0.001) (Supplementary Table S8), the corresponding effect sizes were negligible (Spearman *ρ* = 0.039 for total cost and 0.051 for ICU cost), reflecting the large sample size rather than a clinically meaningful gradient; median ICU LOS also differed slightly across these quartiles (2, 2, 2 and 3 days), so the modest ICU-cost difference cannot be attributed to severity independent of stay duration. The cost gradient observed across risk quartiles in the full cohort therefore appears to be driven principally by the differing proportion of prolonged-stay patients within each quartile (12.9%, 18.3%, 30.9% and 51.2%), rather than by severity captured independently of length of stay.

### 3.6 Sensitivity analysis

Under the 90th-percentile definition, 1,112 patients (14.69%) met the criterion for prolonged ICU stay; this proportion exceeds the nominal 10% because ICU LOS is recorded in whole days and 427 patients had an LOS of exactly 5 days. Reapplying the full pipeline retained ten predictors: pH, absolute monocyte count, BUN, albumin, PaCO_2_, SaO_2_, chloride, fasting plasma glucose, LDH, and age. Seven of the nine predictors from the primary model (pH, BUN, albumin, PaCO_2_, chloride, LDH, age) were retained under both definitions; anterior MI and MCHC were not reselected, while absolute monocyte count, SaO_2_ and fasting plasma glucose entered the model.

Discrimination was comparable to the primary analysis: AUC 0.756 (95% CI: 0.738–0.773) in the training set and 0.715 (95% CI: 0.677–0.753) in the test set, vs. 0.714 and 0.709 respectively in the primary analysis. Bootstrap internal validation gave an optimism-corrected C-index of 0.753. Training-set calibration was good (Brier score 0.109; Hosmer–Lemeshow *P* = 0.533; calibration slope 1.000), but test-set calibration was less satisfactory than in the primary analysis (Brier score 0.114; Hosmer–Lemeshow *P* = 0.030; calibration slope 0.842), with the calibration curve indicating overestimation of risk at the upper end of the predicted-probability range (Supplementary Figure S1). Full results are given in Supplementary Table S9.

## 4 Discussion

Prolonged ICU stay not only increases the risk of nosocomial infections and complications but also directly consumes limited critical care resources and substantially inflates hospitalization costs. However, early prediction tools specifically targeting prolonged ICU stay in AMI populations remain scarce. Previous studies in this area have primarily focused on post-cardiac surgery patients or mixed medical-surgical ICU populations (16–18), and the surgery-related parameters and generic critical illness scores incorporated in those models are not readily transferable to the setting of medical management of AMI. Based on a cohort of 7,571 AMI patients, the present study employed a two-step selection strategy combining Spearman correlation analysis with LASSO regression to identify 9 independent predictors—pH, LDH, BUN, ALB, Anterior MI, Age, PaCO_2_, Cl, and MCHC—from 48 candidate variables obtainable in the early admission period, and constructed a nomogram for predicting prolonged ICU stay. It should be noted that this study adopted a retrospective observational design; the nomogram reflects statistical associations between variables and the outcome rather than causal relationships, and each predictor should be interpreted as a risk marker rather than an intervention target. Nomograms have been widely applied to translate multivariable models into visual tools that can be read directly at the bedside, including in ICU and perioperative risk prediction (19, 20).

Each 100 U/L increase in LDH was associated with a 12% increase in the risk of prolonged ICU stay. As a cytoplasmic enzyme widely distributed in myocardial tissue, serum LDH has long been regarded as a surrogate marker for infarct size. A longitudinal study by Zhang et al. demonstrated that baseline LDH in elderly AMI patients was independently associated with the risk of subsequent cardiac dysfunction and outperformed other myocardial injury biomarkers in predicting ventricular remodeling (21). Each 1 mmol/L increase in BUN was associated with an 8% increase in risk. BUN reflects not only glomerular filtration but also rises disproportionately when cardiac output declines, owing to neurohumoral activation-mediated enhancement of proximal tubular urea reabsorption. Horiuchi et al. demonstrated a stepwise positive association between BUN levels and in-hospital mortality in 3,283 AMI patients, independent of creatinine and estimated glomerular filtration rate (eGFR) (22). A long-term follow-up study by Aronson et al. further confirmed that admission BUN independently predicted long-term cardiovascular mortality in STEMI patients beyond eGFR (23). Each 1 g/L increase in ALB was associated with a 5% reduction in risk. Hypoalbuminemia is a composite reflection of inflammatory burden, nutritional deterioration, and the extent of capillary leakage. González-Pacheco et al. (24) and Plakht et al. (25) independently confirmed in large acute coronary syndrome and AMI cohorts, respectively, that admission albumin levels were independently associated with in-hospital and long-term mortality, supporting its role as a marker of disease severity in AMI patients.

Each 0.1-unit increase in pH was associated with a 54% increase in the risk of prolonged ICU stay, whereas PaCO_2_ was identified as a protective factor. This seemingly paradoxical finding warrants careful interpretation: within the normal or mildly deviated range observed in this cohort, an elevated pH accompanied by a reduced PaCO_2_ may reflect a state of compensatory hyperventilation, which is in fact an indirect signal of more severe hemodynamic compromise. Previous studies have established a close association between early acid-base disturbances and adverse outcomes in AMI (26), and excessive respiratory compensation can be a concomitant manifestation of hemodynamic instability. Each 1 mmol/L increase in Cl was associated with a 3% reduction in risk. Breen et al. identified hypochloremia as an independent predictor of in-hospital mortality in cardiac ICU patients (27), and a 2025 meta-analysis by Wan et al. encompassing 34 studies and 175,021 patients further confirmed that both hypochloremia and hyperchloremia were significantly associated with increased ICU mortality (28). The protective effect of chloride in the present model likely reflects the relatively stable internal milieu represented by higher chloride levels within the normal range.

Each 1 g/L increase in MCHC was associated with a 2% reduction in risk. Huang et al. were the first to report that decreased MCHC was an independent risk factor for in-hospital and 1-year mortality in ICU-admitted AMI patients, postulating that inflammation-driven iron metabolism dysregulation reduces MCHC and impairs red blood cell oxygen-carrying capacity, thereby exacerbating myocardial hypoxia (29). This finding has also been corroborated in patients with heart failure with preserved ejection fraction (30). Age and Anterior MI, as established cardiovascular prognostic factors, also demonstrated independent predictive value in the present model. Anterior MI, by involving a larger area of the left ventricle, leads to more severe left ventricular dysfunction and a higher complication rate (31), making the prolonged need for ICU monitoring clinically intuitive. Although pH, PaCO_2_, BUN and LDH all reflect hemodynamic and end-organ status, they were not statistically collinear, and each contributed independently to the model. That most of the discriminative signal was carried by four of the nine predictors suggests that a more parsimonious nomogram might be nearly as useful at the bedside while being simpler to apply—a possibility worth evaluating prospectively.

The AUCs for the training and test sets were 0.714 and 0.709, respectively, with no statistically significant difference between them. Internal validation with 1,000 bootstrap resamples yielded an optimism-corrected C-index of 0.712 with an optimism estimate of only 0.002 and a calibration slope approaching 1.0, indicating minimal risk of overfitting. Although the AUC falls within the moderate range, it should be considered that the model relies exclusively on routine laboratory parameters available in the early admission period without requiring invasive investigations, and that ICU LOS is inherently influenced by non-patient factors such as clinical decision-making preferences. However, we wish to be explicit about the practical limits this level of discrimination imposes. At the Youden-optimal cutoff, approximately one third of patients who went on to have a prolonged ICU stay were not flagged (sensitivity 64.8%–66.8%), and fewer than half of those flagged as high-risk ultimately met the outcome definition (PPV 43.2%–44.7%). The model therefore cannot serve as a standalone gatekeeping instrument for individual decisions such as ICU admission or discharge timing, and we do not propose it as one. Its more defensible roles are as a rule-out aid at the low-risk end (NPV ≈ 83%; observed prolonged-stay rate of 12.9% in the lowest predicted-risk quartile) and as a group-level triage and resource-planning tool, in which the monotonic risk and cost gradients across predicted-risk quartiles remain informative even where individual predictions carry substantial uncertainty. DCA demonstrated that the model provided a sustained net benefit over threshold probabilities of approximately 15%–80%, covering the principal probability range relevant to clinical decision-making.

These findings do not depend on the specific 75th-percentile threshold: repeating the entire pipeline under a more stringent definition left discrimination essentially unchanged, and seven of the nine predictors were retained under both definitions. The two models were not identical, however—as the outcome was restricted to a more severely ill subset, markers of oxygenation and metabolic stress gained relative predictive weight, an observation that merits further study. Calibration in the test set deteriorated under the stricter definition, consistent with the halving of the event count; discrimination therefore proved more robust to the outcome definition than did absolute probability estimation.

Cost analysis showed that total hospitalization costs in the prolonged group exceeded those in the non-prolonged group by approximately CNY 15,000 (1.49-fold), with bed charges (2.50-fold) and pharmaceutical costs (2.20-fold) showing the most pronounced proportional increases—consistent with prior evidence identifying ICU days as a primary driver of hospitalization costs in AMI patients (32). We acknowledge, however, that this between-group comparison is to a large extent tautological: patients who stay longer necessarily accrue more day-dependent charges. We therefore tested whether the model captured severity over and above stay duration, by comparing costs across predicted-risk quartiles within the non-prolonged group. It did not: cost differences across quartiles were negligible in magnitude (Spearman *ρ* ≤ 0.05; Q4/Q1 ratio 1.02 for total cost), reaching statistical significance only by virtue of sample size. The cost gradient observed across risk quartiles in the full cohort (CNY 32,278 in Q1 to 38,087 in Q4) is therefore best explained by the differing proportion of prolonged-stay patients within each quartile (12.9% to 51.2%), rather than by a severity signal independent of the outcome the model was trained to predict. The economic findings should accordingly be read as a quantification of the expenditure attributable to prolonged ICU stay, which remains directly useful for aggregate bed and budget planning, rather than as independent validation of the model.

This study has several limitations. First, the retrospective design means that the nomogram reflects statistical associations rather than causal relationships. Second, the model was developed in a single centre, where ICU admission and discharge criteria, bed availability, and staffing differ from those elsewhere; we therefore regard multicentre external validation not as a desirable extension but as a prerequisite for any clinical application of this tool, which until then should be considered a locally derived model of uncertain generalisability. Third, no universally accepted threshold for prolonged ICU stay exists; our sensitivity analysis indicates that the core predictors and the level of discrimination are robust to the choice of the 75th vs. the 90th percentile, but the resulting models are not identical, and differing definitions across studies continue to hinder direct comparison. Fourth, the model excludes echocardiographic and coronary lesion characteristics. We acknowledge that left ventricular ejection fraction is among the strongest predictors of outcome in AMI and is available within 24 h in many centres; while this exclusion preserves applicability at the earliest decision point, we accept that the trade-off between early availability and predictive richness may not favour our design, and that a model incorporating ventricular function would plausibly discriminate better. Fifth, laboratory predictors were taken as the first value recorded within 24 h of ICU admission, but the exact sampling time within that window could not be retrieved; as LDH rises progressively after infarction and BUN is sensitive to fluid management, part of the variance these predictors capture may reflect sampling time as well as illness severity. Arterial blood gas parameters were also missing in approximately 24% of patients and were multiply imputed. Sixth, ICU LOS is shaped by non-clinical factors—physician discharge preferences, bed turnover pressure—that no admission-time model can capture, and our model is fixed at a single 24 h time point rather than updating as the patient's trajectory unfolds. Dynamic risk updating using serial measurements, together with multicentre validation, represents the principal direction for future work.

## 5 Conclusion

This study developed and internally validated a nomogram based on nine routinely available early-admission variables for predicting prolonged ICU stay in AMI patients, demonstrating moderate discrimination and good calibration. Supplementary cost analysis established a graded association between nomogram-predicted risk strata and hospitalization expenditures. Its operating characteristics do not support use as a standalone tool for individual admission or discharge decisions, but it may aid group-level risk stratification and ICU resource planning. Multicentre external validation is a prerequisite for clinical application.

## Data Availability

The original contributions presented in the study are included in the article/Supplementary Material, further inquiries can be directed to the corresponding author/s.
