# Supplementary material for: Development and validation of a nomogram for predicting prolonged intensive care unit stay in patients with acute myocardial infarction and analysis of associated hospitalization costs
Source: Front Cardiovasc Med. 2026 Aug 17;13:1900704. doi: 10.3389/fcvm.2026.1900704 (PMC13526946; doi:10.3389/fcvm.2026.1900704)
Supplement: Supplementary file 1 [file DataSheet1.docx]

**Supplementary Material**

**Table S1. Missing value summary for all candidate variables (N = 7,571).**

| **Variable** | **Missing (n)** | **Missing (%)** |
| --- | --- | --- |
| **Demographics** |  |  |
| Age (years) | 0 | 0.00 |
| Sex | 0 | 0.00 |
| BMI (kg/m²) | 321 | 4.24 |
| **Admission** |  |  |
| Admission type | 0 | 0.00 |
| **Myocardial infarction** |  |  |
| Anterior MI | 0 | 0.00 |
| STEMI | 73 | 0.96 |
| Surgery | 0 | 0.00 |
| **Comorbidity** |  |  |
| CCI | 4 | 0.05 |
| **Vital signs** |  |  |
| SBP (mmHg) | 118 | 1.56 |
| DBP (mmHg) | 120 | 1.58 |
| **Complete blood count** |  |  |
| WBC (×10⁹/L) | 111 | 1.47 |
| NEUT% | 111 | 1.47 |
| ANC (×10⁹/L) | 111 | 1.47 |
| ALC (×10⁹/L) | 111 | 1.47 |
| MONO% | 111 | 1.47 |
| AMC (×10⁹/L) | 111 | 1.47 |
| BASO (×10⁹/L) | 111 | 1.47 |
| RBC (×10¹²/L) | 111 | 1.47 |
| Hb (g/L) | 111 | 1.47 |
| PLT (×10⁹/L) | 111 | 1.47 |
| MCHC (g/L) | 112 | 1.48 |
| **Biochemistry** |  |  |
| ALB (g/L) | 735 | 9.71 |
| TP (g/L) | 748 | 9.88 |
| GLOB (g/L) | 743 | 9.81 |
| ALT (U/L) | 114 | 1.51 |
| AST (U/L) | 125 | 1.65 |
| TBIL (μmol/L) | 398 | 5.26 |
| ALP (U/L) | 405 | 5.35 |
| SCr (μmol/L) | 129 | 1.70 |
| BUN (mmol/L) | 129 | 1.70 |
| K (mmol/L) | 167 | 2.21 |
| Na (mmol/L) | 168 | 2.22 |
| Cl (mmol/L) | 168 | 2.22 |
| TCO₂ (mmol/L) | 168 | 2.22 |
| CK (U/L) | 764 | 10.09 |
| CK-MB (U/L) | 369 | 4.87 |
| LDH (U/L) | 435 | 5.75 |
| FPG (mmol/L) | 935 | 12.35 |
| HbA1c (%) | 1993 | 26.32 |
| TG (mmol/L) | 766 | 10.12 |
| LDL-C (mmol/L) | 765 | 10.10 |
| HDL-C (mmol/L) | 765 | 10.10 |
| TC (mmol/L) | 765 | 10.10 |
| **Arterial blood gas** |  |  |
| pH | 1795 | 23.71 |
| PaCO₂ (mmHg) | 1796 | 23.72 |
| PaO₂ (mmHg) | 1801 | 23.79 |
| SaO₂ (%) | 1857 | 24.53 |
| Lactate (mmol/L) | 1849 | 24.42 |

MI, myocardial infarction; STEMI, ST-segment elevation myocardial infarction; CCI, Charlson Comorbidity Index; SBP, systolic blood pressure; DBP, diastolic blood pressure; WBC, white blood cell count; NEUT%, neutrophil percentage; ANC, absolute neutrophil count; ALC, absolute lymphocyte count; MONO%, monocyte percentage; AMC, absolute monocyte count; BASO, basophil count; RBC, red blood cell count; Hb, haemoglobin; PLT, platelet count; MCHC, mean corpuscular haemoglobin concentration; ALB, albumin; TP, total protein; GLOB, globulin; ALT, alanine aminotransferase; AST, aspartate aminotransferase; TBIL, total bilirubin; ALP, alkaline phosphatase; SCr, serum creatinine; BUN, blood urea nitrogen; CK, creatine kinase; CK-MB, creatine kinase-MB; LDH, lactate dehydrogenase; FPG, fasting plasma glucose; TG, triglycerides; LDL-C, low-density lipoprotein cholesterol; HDL-C, high-density lipoprotein cholesterol; TC, total cholesterol.

| **Variable 1** | **Variable 2** | **Spearman ρ** | **ρ with outcome (Variable 1)** | **ρ with outcome (Variable 2)** | **Variable removed** |
| --- | --- | --- | --- | --- | --- |
| Age | CCI | 0.861 | 0.103 | 0.153 | CCI |
| WBC | ANC | 0.958 | 0.071 | 0.069 | ANC |
| NEUT% | ALC | −0.809 | 0.038 | 0.049 | NEUT% |
| MONO% | AMC | 0.862 | 0.063 | 0.090 | MONO% |
| RBC | Hb | 0.911 | 0.050 | 0.054 | RBC |
| AST | CK | 0.934 | 0.180 | 0.156 | CK |
| AST | CK-MB | 0.899 | 0.180 | 0.137 | CK-MB |
| AST | LDH | 0.877 | 0.180 | 0.231 | AST |
| CK | CK-MB | 0.947 | 0.156 | 0.137 | Both previously removed |
| CK | LDH | 0.812 | 0.156 | 0.231 | CK (previously removed) |
| LDL-C | TC | 0.918 | 0.036 | 0.044 | LDL-C |
| PaO₂ | SaO₂ | 0.829 | 0.076 | 0.060 | SaO₂ |

**Table S2. Pairs of mutually collinear candidate variables (|ρ| ≥ 0.8) identified in the training set and the variable removed from each pair.**

The correlation screen was applied only to pairs of mutually collinear predictors. Where |ρ| ≥ 0.8 between two variables, one was retained and the other removed; the correlation with the outcome was used solely as the rule for deciding which member of the pair to retain. Variables not strongly collinear with any other predictor were carried forward irrespective of their marginal association with the outcome. Twelve collinear pairs were identified and ten variables removed (CCI, ANC, NEUT%, MONO%, RBC, CK, CK-MB, AST, LDL-C, SaO₂), leaving 37 variables to enter the LASSO. The threshold of |ρ| ≥ 0.8 follows convention and is not derived from a formal criterion.

**Table S3. Performance metrics of the nomogram in the training and test sets.**

| **Indicator** | **Training set** | **Test set** |
| --- | --- | --- |
| Sample size | 6056 | 1515 |
| Number of events | 1716 | 429 |
| Event rate | 28.34% | 28.32% |
| AUC | 0.714 | 0.709 |
| AUC, 95% CI | 0.700–0.728 | 0.679–0.739 |
| C-index (bootstrap-corrected) | 0.712 | — |
| Brier score | 0.178 | 0.178 |
| Calibration intercept | 0 | −0.041 |
| Calibration slope | 1 | 0.954 |
| Hosmer–Lemeshow χ² | 3.250 | 8.841 |
| Hosmer–Lemeshow P | 0.918 | 0.356 |
| Optimal cutoff (Youden) | 0.264 | 0.279 |
| Sensitivity | 0.668 | 0.648 |
| Specificity | 0.653 | 0.683 |
| PPV | 0.432 | 0.447 |
| NPV | 0.833 | 0.831 |
| Accuracy | 0.657 | 0.673 |

AUC, area under the receiver operating characteristic curve; CI, confidence interval; PPV, positive predictive value; NPV, negative predictive value. The bootstrap-corrected C-index was obtained from 1,000 resamples.

**Table S4. Variance inflation factors for the nine predictors in the final multivariable logistic regression model.**

| **Predictor** | **VIF** | **Tolerance (1/VIF)** |
| --- | --- | --- |
| BUN | 1.339 | 0.747 |
| PaCO₂ | 1.321 | 0.757 |
| pH | 1.299 | 0.770 |
| ALB | 1.288 | 0.776 |
| LDH | 1.233 | 0.811 |
| Age | 1.217 | 0.822 |
| Anterior MI | 1.215 | 0.823 |
| Cl | 1.149 | 0.870 |
| MCHC | 1.085 | 0.922 |

VIF, variance inflation factor. A VIF ≥ 5 was taken to indicate potentially problematic collinearity and a VIF ≥ 10 severe collinearity. All values were below 1.34, including those for pH and PaCO₂, which are physiologically linked through the Henderson–Hasselbalch relationship.

**Table S5. Performance of nested reduced models compared with the full nine-predictor model.**

| **No. of predictors** | **Predictors added** | **Training AUC** | **Test AUC (95% CI)** | **AIC** | **DeLong P vs full model** | **LRT P vs full model** |
| --- | --- | --- | --- | --- | --- | --- |
| 2 | LDH, BUN | 0.685 | 0.692 (0.661–0.722) | 6705.9 | 0.067 | <0.001 |
| 3 | + ALB | 0.696 | 0.699 (0.669–0.730) | 6644.0 | 0.217 | <0.001 |
| 4 | + pH | 0.707 | 0.710 (0.680–0.740) | 6573.9 | 0.891 | <0.001 |
| 5 | + MCHC | 0.709 | 0.711 (0.681–0.741) | 6556.5 | 0.702 | <0.001 |
| 6 | + Anterior MI | 0.711 | 0.708 (0.678–0.737) | 6538.6 | 0.645 | <0.001 |
| 7 | + Age | 0.712 | 0.707 (0.678–0.737) | 6527.4 | 0.490 | <0.001 |
| 8 | + Cl | 0.713 | 0.706 (0.676–0.736) | 6520.3 | 0.165 | 0.001 |
| **9 (full model)** | **+ PaCO₂** | **0.714** | **0.709 (0.679–0.739)** | **6511.5** | **Reference** | **Reference** |

Predictors were added in descending order of their Wald χ² statistic in the full model (LDH > BUN > ALB > pH > MCHC > anterior MI > age > Cl > PaCO₂). AIC, Akaike information criterion; LRT, likelihood-ratio test. DeLong tests compare test-set AUC against the full model; likelihood-ratio tests compare model fit in the training set against the full model. A four-predictor model achieved a test-set AUC statistically indistinguishable from the full model (DeLong P = 0.891), although the additional predictors improved model fit (LRT P < 0.001; AIC declining monotonically).

**Table S6. Comparison of hospitalization cost components between the prolonged and non-prolonged ICU stay groups.**

| **Cost category** | **Prolonged ICU LOS (n = 2,145)** | **Non-prolonged ICU LOS (n = 5,426)** | **Median difference (CNY)** | **P value** |
| --- | --- | --- | --- | --- |
| Total hospital cost | 45,457 (30,543, 64,131) | 30,408 (22,160, 44,392) | 15,049 | <0.001 |
| ICU cost | 40,494 (26,794, 56,436) | 25,519 (19,352, 38,147) | 14,975 | <0.001 |
| Medication | 3,540 (2,177, 6,603) | 1,612 (907, 2,725) | 1,929 | <0.001 |
| Examination | 4,225 (2,900, 6,397) | 2,736 (1,484, 3,508) | 1,489 | <0.001 |
| Laboratory | 4,675 (4,013, 5,943) | 3,283 (2,856, 3,710) | 1,392 | <0.001 |
| Surgery | 330 (0, 330) | 330 (0, 330) | 0 | <0.001 |
| Material | 16,164 (6,169, 28,447) | 9,607 (4,734, 21,262) | 6,557 | <0.001 |
| Treatment | 7,337 (6,072, 8,659) | 6,015 (5,344, 7,311) | 1,322 | <0.001 |
| Bed | 330 (264, 396) | 132 (132, 198) | 198 | <0.001 |
| Nursing | 737 (604, 1,006) | 355 (268, 443) | 382 | <0.001 |
| Other | 470 (408, 627) | 290 (224, 339) | 180 | <0.001 |

Costs are presented as median (interquartile range) in Chinese Yuan (CNY). P values were derived from Mann–Whitney U tests.

**Table S7. Hospitalization and ICU costs stratified by nomogram-predicted risk quartile (full cohort).**

| **Risk quartile** | **n** | **Mean predicted probability** | **Observed prolonged LOS rate (%)** | **Total cost, CNY, median (IQR)** | **ICU cost, CNY, median (IQR)** |
| --- | --- | --- | --- | --- | --- |
| Q1 (low) | 1,893 | 0.129 | 12.9 | 32,278 (22,035, 44,759) | 25,863 (18,844, 38,724) |
| Q2 | 1,893 | 0.204 | 18.3 | 32,969 (22,988, 47,919) | 27,270 (20,306, 41,856) |
| Q3 | 1,892 | 0.293 | 30.9 | 34,744 (23,725, 51,993) | 28,650 (21,016, 43,904) |
| Q4 (high) | 1,893 | 0.508 | 51.2 | 38,087 (26,488, 54,798) | 33,590 (23,501, 50,161) |

Costs are presented as median (interquartile range) in Chinese Yuan (CNY). Risk quartiles were defined by the nomogram-predicted probability of prolonged ICU stay.

**Table S8. Hospitalization costs across nomogram-predicted risk quartiles within the non-prolonged ICU stay group (n = 5,426).**

| **Risk quartile** | **n** | **Mean predicted probability** | **ICU LOS, days, median (IQR)** | **Total cost, CNY, median (IQR)** | **ICU cost, CNY, median (IQR)** |
| --- | --- | --- | --- | --- | --- |
| Q1 (low) | 1,649 | 0.129 | 2 (2, 3) | 29,574 (21,366, 42,477) | 24,082 (18,428, 36,649) |
| Q2 | 1,546 | 0.203 | 2 (2, 3) | 31,133 (22,290, 45,485) | 25,587 (19,697, 38,994) |
| Q3 | 1,308 | 0.291 | 2 (2, 3) | 31,081 (22,284, 46,211) | 25,897 (19,943, 38,953) |
| Q4 (high) | 923 | 0.477 | 3 (2, 3) | 30,070 (22,981, 43,812) | 26,521 (19,988, 38,713) |
| Q4/Q1 ratio | — | — | — | 1.02 | 1.10 |
| Kruskal–Wallis P | — | — | — | <0.001 | <0.001 |
| Spearman ρ (predicted probability vs cost) | — | — | — | 0.039 | 0.051 |
| Spearman P | — | — | — | 0.004 | <0.001 |

Risk quartiles were defined using the cut-points derived from the full cohort. Costs are presented as median (interquartile range) in Chinese Yuan (CNY). Although the Kruskal–Wallis tests reached statistical significance, the corresponding effect sizes were negligible (Spearman ρ ≤ 0.05) and the Q4/Q1 cost ratios were close to unity, indicating that the statistical significance reflects the large sample size rather than a clinically meaningful cost gradient. Median total cost was not monotonic across quartiles. Median ICU length of stay also differed slightly across quartiles (2–3 days), so the modest difference in ICU cost cannot be attributed to severity independent of stay duration.

**Table S9. Sensitivity analysis: model performance under the 90th-percentile definition of prolonged ICU stay (≥5 days).**

| **Indicator** | **Training set** | **Test set** |
| --- | --- | --- |
| Sample size | 6056 | 1515 |
| Number of events | 889 | 223 |
| Event rate | 14.68% | 14.72% |
| AUC | 0.756 | 0.715 |
| AUC, 95% CI | 0.738–0.773 | 0.677–0.753 |
| C-index (bootstrap-corrected) | 0.753 | — |
| Brier score | 0.109 | 0.114 |
| Calibration intercept | 0 | −0.242 |
| Calibration slope | 1 | 0.842 |
| Hosmer–Lemeshow χ² | 7.033 | 17.016 |
| Hosmer–Lemeshow P | 0.533 | 0.030 |
| Optimal cutoff (Youden) | 0.153 | 0.128 |
| Sensitivity | 0.646 | 0.682 |
| Specificity | 0.747 | 0.662 |
| PPV | 0.305 | 0.258 |
| NPV | 0.925 | 0.923 |
| Accuracy | 0.732 | 0.665 |

The entire analytic pipeline (Spearman correlation screening, LASSO selection at λ1se, multivariable logistic regression and bootstrap internal validation) was repeated under this alternative outcome definition, holding the 8:2 split, the random seed and all preprocessing steps constant. Ten predictors were retained (pH, absolute monocyte count, BUN, ALB, PaCO₂, SaO₂, Cl, fasting plasma glucose, LDH and age), seven of which were shared with the primary nine-predictor model. Abbreviations as in Table S3.

**Figure S1. Model performance under the 90th-percentile definition of prolonged ICU stay (≥5 days). (A) Receiver operating characteristic curves for the training and test sets. (B) Calibration curves for the training and test sets.**
